# Outcomes of Quadriceps and Patellar Tendon Repairs Augmented With Polyester Tapes

**DOI:** 10.7759/cureus.80856

**Published:** 2025-03-19

**Authors:** Cameron Kennedy, Gunesekeran Kumar

**Affiliations:** 1 Orthopaedics, Aintree University Hospital, Liverpool, GBR; 2 Trauma and Orthopaedics, Aintree University Hospital, Liverpool, GBR

**Keywords:** patella tendon, quadriceps tendon, retrospective cohort, tendon repair, tension banding

## Abstract

Introduction: Rupture of the quadriceps tendon and patellar tendon (QT/PT) is an infrequent injury that displays a bimodal distribution, typically in low-energy injuries in elderly patients and sporting injuries in younger patients. Independent risk factors for QT/PT rupture include renal failure, obesity, steroid or antibiotic use, old age, and diabetes. Prompt repair restores the mobility of the extensor mechanism of the leg and failure can lead to significant morbidity. Augmenting QT/PT repair with polyester tapes could assist in improving the strength of the repair. The purpose of this study was to assess the outcomes of QT/PT repair augmentation with polyester tape.

Methods: A single-centre retrospective cohort analysis was conducted during the period from January 2016 to December 2023 on patients who underwent QT/PT rupture repairs augmented with polyester tapes. Data collected included the mechanism of injury, associated risk factors, unilateral or bilateral injuries, time to surgery, complications, range of movements, outcomes, and the duration of follow-up. Operative outcomes were recorded as poor, satisfactory, good, or excellent and depended on the patient's range of movement, ability to weight bear, return to pre-trauma level of fitness, and need for revision. Multiple regression analysis was performed to assess the influence of risk factors on the incidence of QT/PT rupture.

Results: Over the seven-year period, 76% (n = 69; quadriceps tendon (QT) = 22, patellar tendon (PT) = 43, and bilateral = 4) of patients with QT/PT repairs augmented with polyester tapes met the inclusion criteria. The most common mechanisms of injury were a fall downstairs (n = 52) and a twisting injury in sports (n = 11). The average postoperative follow-up was 10 months. A total of 88% achieved excellent (n = 45) and good (n = 11) outcomes following QT/PT repair, and five patients failed to attend follow-up. Having more than two significant risk factors (p < 0.05) and a high impact mechanism (p < 0.05) of injury were the most significant predictors for poor functional outcomes. Of the eight patients with poor outcomes, six had two or more risk factors. All four patients (three with infection and one with PT laxity) who underwent further surgery had two or more risk factors.

Discussion and conclusion: Augmenting QT/PT repairs with polyester tapes did not have any adverse effects and is associated with good to excellent outcomes. Risk factors for QT/PT ruptures are also associated with postoperative complications and poor outcomes.

## Introduction

Rupture of the extensor mechanism tendons of the knee is a rare and debilitating condition that requires surgical intervention. Prompt repair of the quadriceps tendon and patellar tendon (QT/PT) mechanism restores the mobility of the extensor mechanism of the leg and can prevent progressive degenerative changes [1]. Failure can lead to significant morbidity in the long term [1].

The pathology has a bimodal distribution [2]. QT/PT rupture in younger patients is associated with direct trauma [1-3]. In contrast, the aetiology of QT/PT ruptures in patients older than 40 years is often a consequence of progressive degenerative change secondary to systemic disease and lifestyle factors [4,5]. Reported risk factors associated with QT/PT rupture include obesity, diabetes mellitus (DM), chronic renal failure, concurrent steroid use, smoking, connective tissue disorders, and quinolones [5].

The technique most frequently described for QT/PT repair places a continuous interlocking suture through the distal end of the tendon and secures with drill holes into the patella. Low re-rupture rates following this technique have been reported (mean = 2%, range = 0-8.3%) [6]. However, poor functional outcomes for patients reported in the literature range from 10% to 22% [6].

In recent literature, augmentation techniques have demonstrated favourable outcomes in the management of acute QT/PT rupture [7]. Augmentation techniques are widely employed in orthopaedic surgery, and in QT/PT rupture repair, they are reported to decrease strain across the repair, which could result in earlier knee motion [8,9].

In this study, we aim to assess the outcomes of QT/PT repairs that have been augmented with polyester tape. Additionally, we aim to identify whether outcomes are significantly affected by preoperative and patient factors to help dictate and guide future practice.

## Materials and methods

Data collection

A single-centre retrospective cohort analysis was conducted on patients who underwent QT/PT repair at Aintree University Hospital. Data were collected during the period from January 2016 to December 2023. Only patients who had QT/PT repairs augmented with 30 mm wide polyester tapes were included in the dataset.

In all cases, a standardised, non-augmented technique was performed initially to repair the patellar tendon (PT) or quadriceps tendon (QT).

Two nonabsorbable sutures (Ti-Cron or Ethibond) were passed through and fixated to the quadriceps or patellar tendon by applying a Krackow stitch. This leaves one medial, one lateral, and two middle sutures exiting the free edge of the ruptured tendon. The patella is prepared by drilling medial, middle, and lateral longitudinal holes through the anterior surface. The two medial sutures pass through the middle hole, with the middle and lateral sutures passing through their respective holes. With the knee in full extension, the medial middle suture is tied to the medial suture and the lateral middle suture is tied to the lateral suture. Following the standard QT/PT tendon repair, the approach was augmented with polyester tape.

A 30 mm x 800 mm polyester tape is passed through the proximal quadriceps tendon and then both ends are crossed over the anterior surface of the patella to form a figure of eight. The medial end of the polyester tape is passed through a tunnel created in the tibial tuberosity, with both ends then anchored to the lateral tibial metaphysis by knot tying the free ends of the polyester tape, and the excess tape was then cut.

Inter-operative variability of technique was reduced by only including data from a single surgeon’s patient dataset. Patient information was anonymised and collected from a patient’s electronic health record and recorded in an Excel database (Microsoft Corporation, Redmond, WA).

Preoperative data collected included the mechanism of injury, associated risk factors, unilateral or bilateral injuries, and time to surgery.

Postoperative outcomes were recorded by reviewing clinic letters and physio reports. Operative outcomes were recorded according to grades I-IV of the Clavien-Dindo classification [10]: grade I: no change to the routine postoperative course; grade II: requires a change in outpatient management; grade III: requires invasive surgical management, but no long-term morbidity; grade IV: long-term morbidity or a life-threatening complication.

Data used to decide the Clavien-Dindo classification included arthritis, joint laxity, re-rupture, range of movement (RoM), revision surgery, and duration of follow-up.

Data analysis

A multiple regression analysis was performed to assess the influence of risk factors on the incidence of QT/PT rupture and patient outcomes.

## Results

Over seven years, 64 patients underwent QT/PT repair (QT = 22, PT = 43, and bilateral = 4). Patients in this study had a mean age of 55 years. More male patients underwent QT/PT repair compared to females (male = 46; female = 18). Patients were followed up over an average of 11 months (range = 0.5-72). Five patients were lost to follow-up (Table 1).

**Table 1:** Demographic data.

Characteristics	Number
Male	46
Female	18
Age (mean)	55
Diabetic	4
Non-diabetic	62
Obese	15
Not obese	49

A total of 83% achieved grade I (n = 43) and grade II (n = 10) outcomes following QT/PT repair, and five patients required revision. All patients (n = 5; infection = 3 and PT laxity = 2) who underwent revision had two or more risk factors associated with poor outcomes. No patients reported a re-rupture of the QT/PT following augmented repair with polyester tape.

Having more than two significant risk factors (p < 0.05, p = 0.0002, r² = 0.22) is a significant predictor for poor functional outcomes. No significant difference in patient outcomes was reported between the use of Ethibond and Ti-Cron sutures (p > 0.05).

Similarly, patients who suffered bilateral tendon rupture did not have worse outcomes when compared to patients with unilateral QT/PT rupture (p > 0.05) (Tables 2, 3).

**Table 2:** Multiple regression analysis of the impact of preoperative factors on patient outcomes following polyester tape tension banding.

	Coefficients	Standard error	t stat	P-value
Sex	-0.032	0.09	-0.362	0.719
Smoking	0.258	0.405	0.638	0.526
Age > 60	0.066	0.263	0.252	0.802
Obesity	-0.472	0.328	-1.439	0.156
Side	-0.473	0.441	-1.074	0.287
High-energy mechanism	0.796	0.464	1.716	0.092
>2 risk factors	0.513	0.146	3.505	0.001

**Table 3:** Multiple regression analysis of the impact of preoperative factors on patient outcomes following polyester tape tension banding.

R²	0.23
Adjusted R²	0.14
n	64
F value	2.41
F significance	0.03

## Discussion

The most significant finding demonstrated in our study was that augmentation of QT/PT repair with polyester tape leads to good patient outcomes, low recurrence, and low rates of revision in patients with one or fewer risk factors.

Biomechanical studies in cadaveric specimens have demonstrated the benefit of augmentation when compared to traditional techniques in allowing for a higher maximal load following QT/PT repair [9,11,12]. Augmenting with polyester tape and wires have demonstrated comparable outcomes [7].

A biomechanical study in patients who underwent augmented QT/PT repair also demonstrated that there was no significant difference in strength measurement in knee extension with the contralateral leg [13]. However, the initial data are limited by study size (n = 7). In comparison to the study conducted by Hinz et al., this study was unable to record standardised follow-up outcomes for all of our patients and the data collected on patient outcomes were predominantly qualitative [13]. This was a result of our data and study design being conducted retrospectively.

Gilmore et al.'s systematic review of modern QT/PT repair techniques and their impact on patient outcomes strengthens the evidence outlined in the biomechanical studies, reporting that the best patient outcomes were reported by those who had undergone augmented repair [7].

The 0% re-rupture rate recorded in this report is comparable to the 2% reported by Gilmore et al. [7]. However, the difference when compared to the results from a non-augmented technique cannot be reported as significant (p > 0.05) [6].

Our most frequently reported cause of failure and poor patient outcomes was a deep postoperative infection (4.6%). The risk of infection is theoretically higher in patients with augmented QT/PT repair due to the introduction of additional synthetic material into the wound. Initially, it was thought that polyester augmentation tape was associated with higher risks of infection [14]. However, more recent studies have challenged this [15].

In comparison to non-augmented tendon repair, the infection rate is comparable with the results in our study. The large systematic review (n = 3442) of post QT/PT repair conducted by Lewis et al. reported infection rates to be 6.3% in patients undergoing PT repair and 2.6% in QT repair [16]. DM was reported to be a significant risk factor for postoperative infection (p = 0.005) [16]. Similarly, two out of three patients in our study who required revision due to a postoperative infection had a past medical history of DM. Consideration of the use of augmentation in diabetic patients should be considered to potentially reduce the risk of postoperative infection.

Limitations

The short follow-up time (mean = 11 months) of the patients limits the validity of the results of this study and we are unable to comment on the durability of the polyester tapes. Additionally, a standardised method of conducting postoperative reviews of the patient would be required to accurately record outcomes in quality of life and functionality. Additionally, five patients were lost to follow-up, which could introduce a reporting bias.

Finally, on review of the literature reporting outcomes of augmented and non-augmented repairs of QT/PT rupture, different studies apply separate classification systems to record postoperative outcomes. As a result, direct comparison of outcomes is limited due to a lack of a non-standardised classification for patients following QT/PT ruptures.

## Conclusions

Augmentation of QT/PT reconstruction with polyester tape has shown to have excellent outcomes in patients with QT/PT ruptures with no recorded cases of re-rupture. Postoperative infection rates in diabetic patients following augmented repair are higher than in non-diabetic patients and the use of polyester tapes in DM patients should be restricted.
